# Differential surface plasmon polaritons transmission line with controllable common mode rejection

**DOI:** 10.1038/s41598-017-03242-6

**Published:** 2017-06-07

**Authors:** Xue-Feng Zhang, Jian-Xin Chen, Rui-Feng Gao, Chen Xu, Zhi-Hua Bao

**Affiliations:** School of Electronics and Information, Nantong University, Nantong, 226019 China

## Abstract

In this paper, a spoof surface plasmon polarions (SPPs) transmission line is designed by patterning thin metal film in open-cross shape arranged in array. Numerical simulations show the proposed open-cross array can support spoof SPPs with enlarged propagation constant and hence enhanced confinement at metal/dielectric interface as compared to the reported ultra-thin plasmonic waveguide with the rectangular groove or solid-cross. Furthermore, a differential transmission line pair is built with such two close plasmonic arrays. A narrow metal strip locates at the symmetrical plane of the two SPPs waveguides and acts as a resonator to realize common-mode rejection at specific frequency. The notch frequency for common mode can be adjusted by tuning the metal strip length of the resonator while differential mode propagation remains unaffected. Both simulated and experimental results with good agreement are given to verify the proposed idea.

## Introduction

Recently, surface plasmon polaritons (SPPs) have attracted great attentions due to the feature of subwavelength confinement, field enhancement, and slow-wave^1–3^. The electromagnetic (EM) filed is tightly bound at a metal/dielectric interface because of the negative permittivity of metals at visible frequency^3^. They are finding an ever increasing number of applications in the traditional domains of surface characterization, biomedical sensing, and near-field microscopy^4, 5^. However, the SPPs cannot be excited since metals behave like perfect electric conductors (PEC) in the frequency range of microwave to terahertz. To mimic the nature of SPPs in this spectrum, spoof (or designer) SPPs have been proposed by etching a metal surface with periodic grooves or holes^6, 7^, which provides another freedom in controlling the electromagnetic (EM) propagation by designing the geometry of the periodic corrugations. Early spoof SPPs are usually designed in 3D geometry thus limiting their applications to compact planar circuits. Shen *et al*.^8–10^ proposed an ultra-thin plasmonic structure, which is formed by patterning the metal film on an ultra-thin and flexible dielectric, to confine and guide SPPs. Afterwards, various ultra-thin plasmonic structures have been developed to enhance the confinement on SPPs and the transmission efficiency of the SPPs^11, 12^. As compared with the traditional microstrip, the ultra-thin plasmonic structure exhibits a smaller transmission loss in the microwave frequencies and thus has promising applications in integrated devices or circuits for performance enhancement^13–15^.

In the past few years, differential topology is widely adopted in constructing microwave/millimeter-wave circuits due to inherent superior signal-to-noise ratio and high immunity to environmental noise and electromagnetic interference^16–19^. Most of differential circuits have been constructed with conventional planar transmission lines (T-lines) such as microstrip and coplanar waveguide (CPW) and only few is concerned with plasmonic T-lines^20^. Recent results show the advantage of crosstalk suppression of SPP T-lines facilitates the improvement of signal integrity in circuits on PCB and chip level^20, 21^. However, to the author’s best knowledge, the common mode suppression has not been mentioned so far. Indeed, the common mode suppression is desired for practical applications in microwave circuits.

In this work, a new plasmonic structure with open-cross unit cell is proposed to enhance the confinement of EM field, and then a single low loss SPPs T-line is constructed and its transmission characteristics is simulated and validated by experiment. Based on this, a low-loss differential transmission line pair is constructed to explore the transmission characteristics for both the differential and the common mode of the SPPs T-lines. Meanwhile, a narrow metal strip at the symmetrical plane of the two SPPs T-lines is introduced as a bandstop structure for the common mode while it has no effect on the differential mode. The notch of the common mode can be adjusted to the desired frequency by tuning the strip length. In his work, controllable common mode suppression is demonstrated in differential SPPs T-line pair.

## Results

### Unit cell design and dispersion relations of the designed plasmonic structure

**Figure 1:**
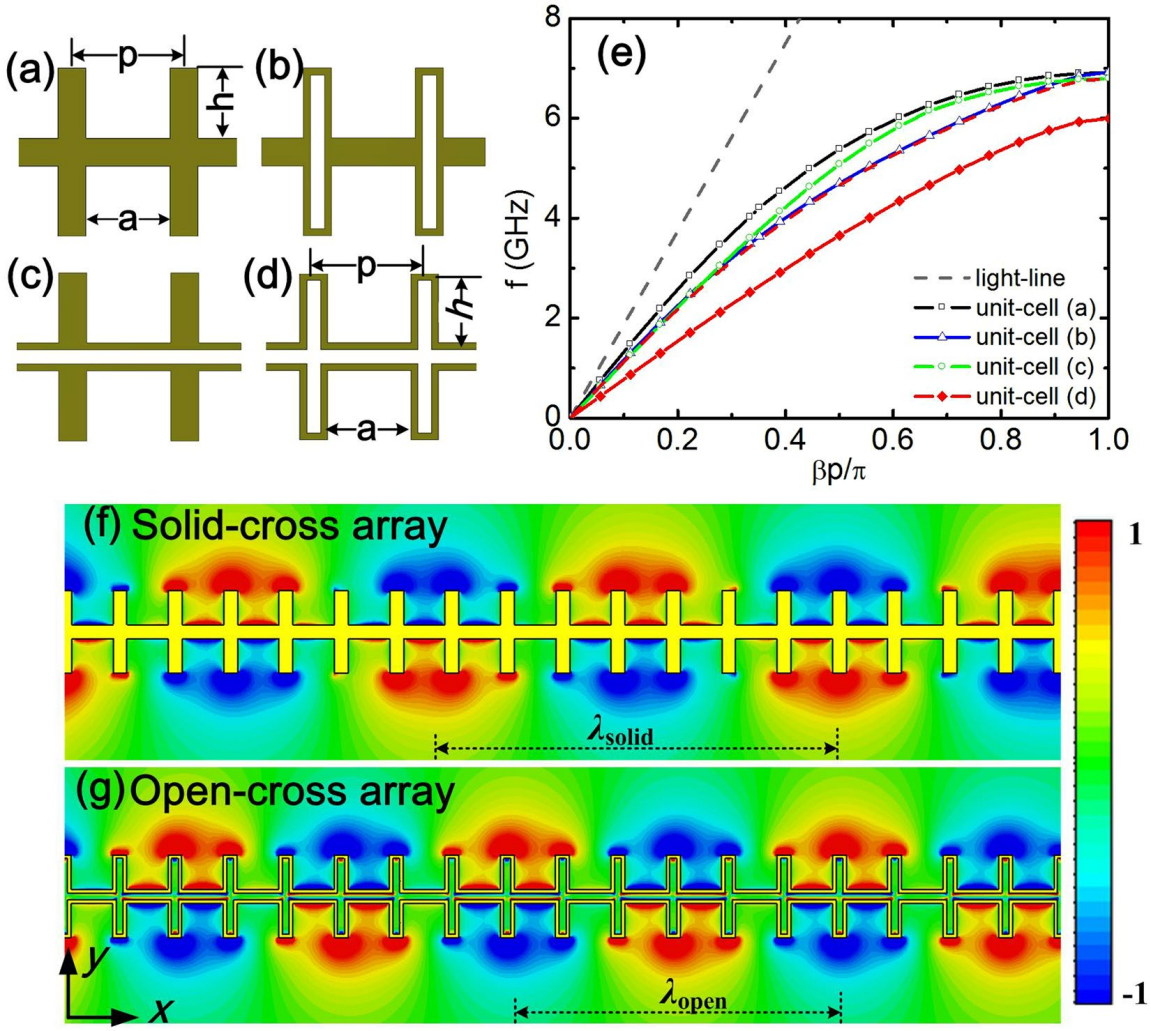
Schematic shapes of unit cell to construct plasmonic arrays: **(a)** solid-cross; **(b)** cross with window; **(c)** double solid-cross; **(d)** open-cross. The unit cells are formed by patterning 0.018 mm-thick metal film on dielectric substrates with a thickness of 1.524 mm and a permittivity of 3.55. **(e)** Dispersion relations of the fundamental modes for unit cells with shapes from. **(a)** to **(d)** Distribution of the real part of electric field *E*_y_ at frequency of 3.5 GHz for a solid-cross array **(f)** and an open-cross array **(g)**.

Figure 1(a) shows the conventional rectangular (or solid-cross) shape unit cell that is usually used to construct plasmonic waveguide/transmission line. In this work, the center of solid-cross is deliberately dug out to enlarge propagation constant. The derived unit cells with other shapes are shown in Fig. 1(b–d). The dimensions of these unit cells are as follows: the periodicity *p* = 8 mm, the groove depth *h* = 6 mm, and gap *a* = 6 mm. The center slot of unit cells of (b)–(d) is 1 mm. All of the above unit cells are formed by patterning 0.018 mm-thick copper film on Roger 4003 dielectric substrates with a thickness of 1.524 mm and a permittivity of 3.55. Under the PEC approximation, the dispersion relations of the unit cells in periodicity boundary are analyzed using the eigen-mode solver of commercial full-wave software, CST Microwave studio. Figure 1(e) shows the simulated dispersion curves of fundamental modes for the unit cells of (a)–(d). All of them deviate significantly from the light line. The asymptotic frequency is dropped from 7 GHz to 6 GHz when the unit cell shapes evolves from (a) to (d). In among, the proposed open-cross unit cell in Fig. 1(d) exhibits the lowest asymptotic frequency and largest prorogation constant *β*. It is known that the EM field of plasmonic surface wave is confined mainly at the interface of corrugated metal strip and dielectric, and exponential decays away from the interface. The confinement of plasmonic surface wave is dependent on the exponential decay constant *α*, which is defined by $\alpha =1/\sqrt{{\beta }^{2}-{{k}_{0}}^{2}}$, where *β* is the prorogation constant and *k*_0_ the wave number in free space. Figure 1(e) and (f) show the simulated distribution of electric field *E*_y_ (real part) at frequency of 3.5 GHz for the open-cross array and the solid-cross array respectively. The open-cross arrays support a shorter guided wavelength (*λ*_open_ ≈ 48 mm) than the solid-cross ones (*λ*_solid_ ≈ 60 mm), which means a larger *β* and *α*, or in other words, tighter confinement on spoof SPPs. Therefore, the enhancement of confinement on spoof SPPs can be achieved by using the proposed open-cross unit cell.

### Straight and differential plasmonic transmission line

**Figure 2:**
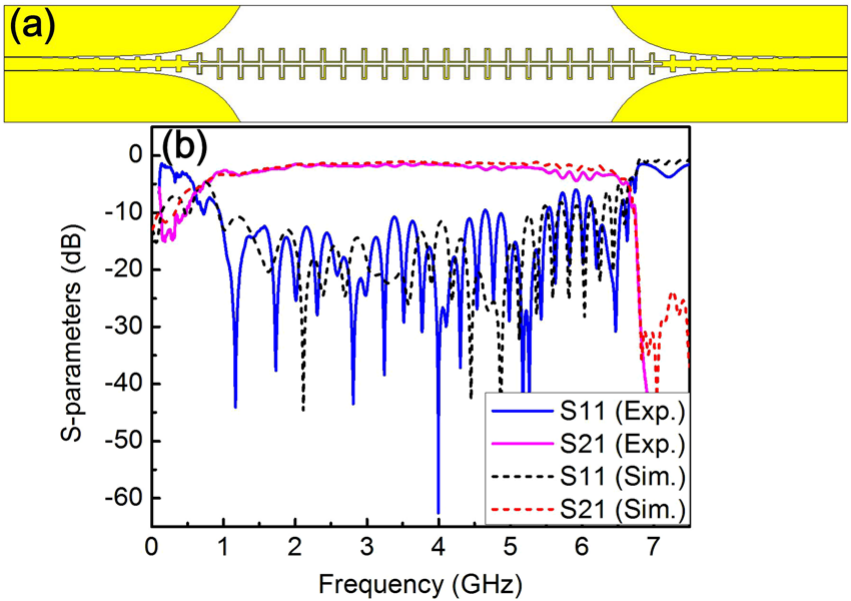
(**a**) Schematic of the straight SPPs T-line constructed with open-cross unit cell, **(b)** comparison of the curves of simulated and experimental scattering parameters.

To explore the transmission properties of open-cross array, a two-port straight spoof SPPs T-line with the configuration, as shown in Fig. 2(a), are fabricated by patterning the 0.018 mm-thicknenss copper film on 1.524 mm-thickness Roger 4003 substrate. As reported in refs 22 and 23, a mode converter is necessary to realize a smooth conversion between guide wave (quasi-TEM or TEM mode) to SPPs (the bound TM mode). In this work, mode converters are used to connect the CPW T-lines with SPPs T-lines to feed in/out EM energy, where the gradient grooves is designed to match the momentums of the quasi-TEM mode in CPW and the bound TM mode in plasmonic waveguide, and the flaring grounds to match impedences of the two T-lines^23^. Figure 2(b) shows the measured scattering parameters (S-parameters) for the straight sample performed on a four-port Agilent N5230C PNA-L network analyzer, along with simulated results for comparison, in which the measured reflection coefficient (*S*11) is less than −10 dB in frequency range of 0.95~5.5 GHz and insertion loss is about 1 dB ± 0.1 from 3.2 GHz to 4 GHz. The high return loss in the low frequency range (<0.9 GHz) results from the incomplete excitation of surface mode and thus leads strong scattering on incoming EM wave. It is also noted that the simulated transmission performance is a little better than the measured one, which can be attributed to the extra loss introduced by the connectors for the test.

**Figure 3:**
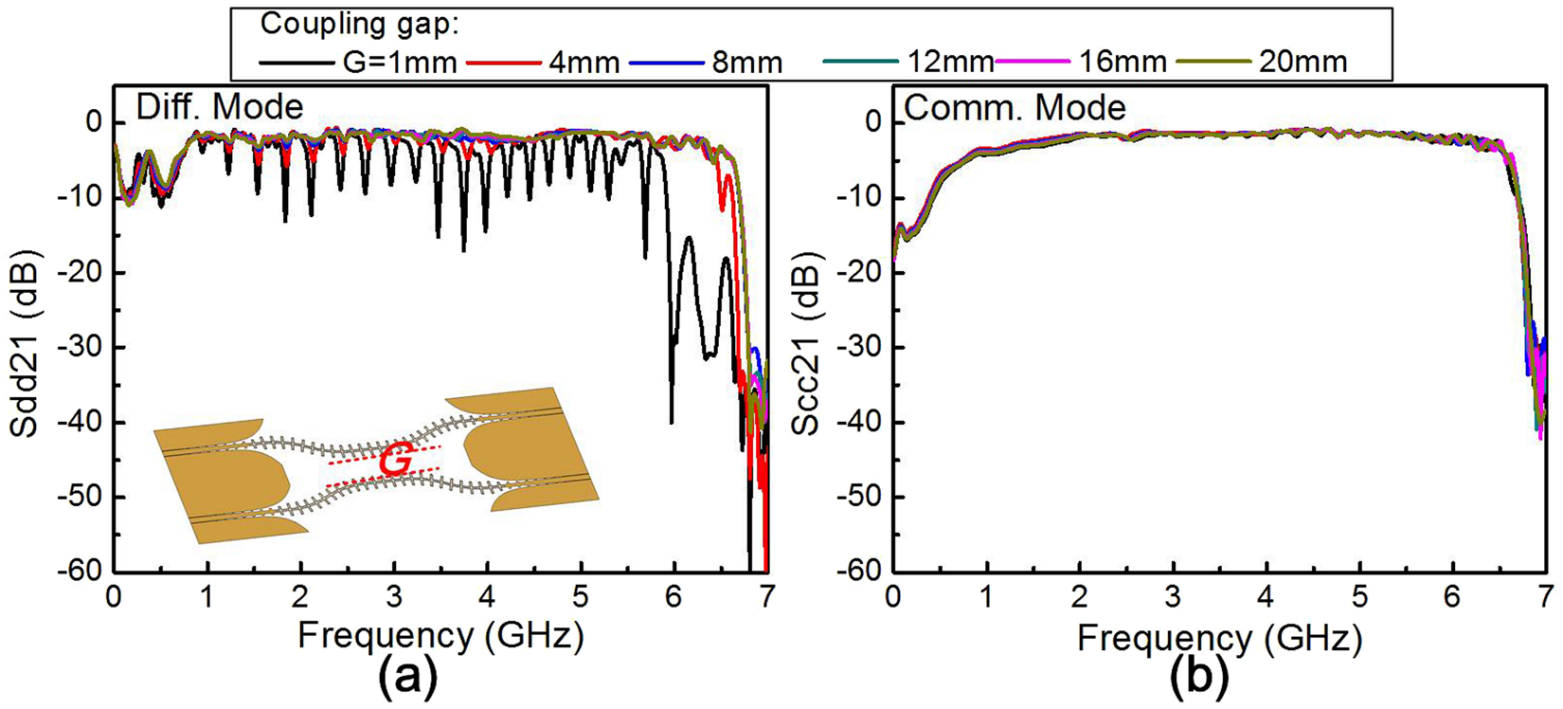
The effect of the coupling gap on transmission coefficient of **(a)** differential mode (*S*_dd_21) and **(b)** common mode (*S*_cc_21).

A pair of differential SPPs T-lines is designed and fabricated by using the proposed open-cross unit cell. The schematic of the couple SPPs T-lines is shown as the inset of Fig. 3(a), where the center parts of two adjacent plasmonic arrays with a separation *G* form a coupling section. Numerical simulations on this differential pair are carried out to investigate the coupling characteristics. In most cases, the differential mode transmission coefficient *S*_dd_21 is almost the same as common mode transmission coefficient *S*_cc_21 in the frequency range of interest. It means little interference between the two adjacent SPPs T-lines due to the high confinement of EM field in SPPs structures. However, as the two T-lines are close enough, such as the coupling gap being less than 8 mm, the interactional coupling takes place. It is noted that a series of notches in the *S*_dd_21curves while the *S*_dd_21 curves remaining unchanged, as shown in Fig. 3(a),(b). This multiple narrowband transmission resonance arises from the interference between the forward propagating and the backward propagating waves in the two closely placed SPPs T-lines^21^, which deteriorates the signal integrity. Consequently, a little larger coupling gap of 10 mm was applied in our prototype sample fabrication.

**Figure 4:**
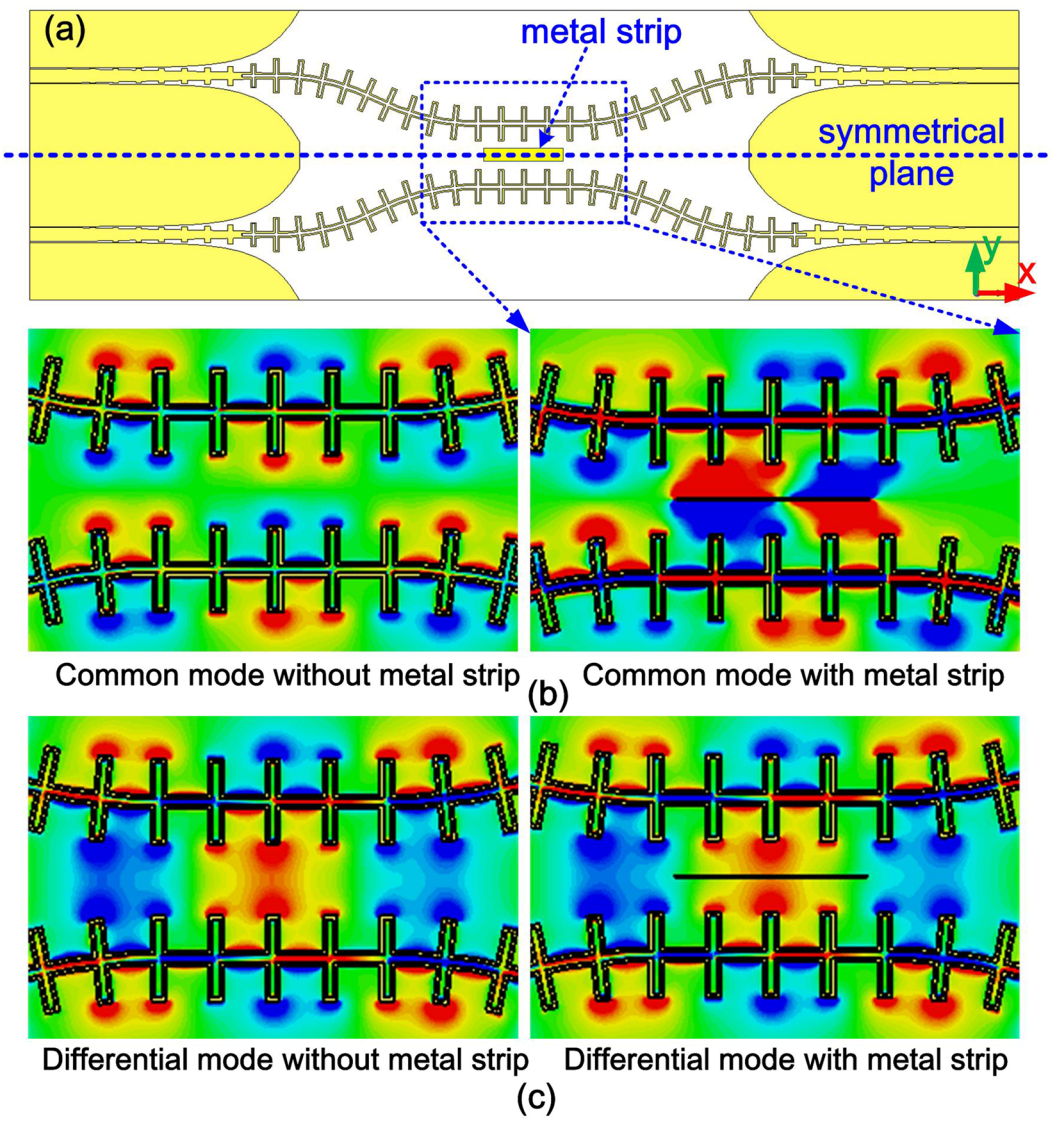
(**a**) Schematic of the differential plasmonic transmission lines. Comparisons of distribution of electric field *E*_y_ at frequency of 3.5 GHz for common mode **(b)** and **(c)** for differential mode. The left figures of **(b)** and **(c)** are results without metal strips and right ones with metal strips.

To suppress common mode propagation in the differential pair, a narrow metal strip paralleled to the plasmonic arrays is introduced at the center of the symmetrical plane. As shown in Fig. 4(a), the differential structure is symmetrically configured with two adjacent plasmonic arrays, and the center parts of them are left a 10 mm gap. A narrow metal strip locating at the center of the symmetrical plane is used to prevent the common mode propagation at the resonant frequency of the narrow strip.

The basic idea of introducing a metal strip at the center of the symmetrical plane is that the symmetrical plane functions as a virtual ground when the differential mode transmits along the SPPs pair, and thus a narrow loaded strip has no effect on the differential mode. While as for the common mode, a magnetic wall locates along the symmetrical plane, the EM field of the common mode is disturbed by the loaded strip, resulting in common-mode suppression at specific frequency (corresponding to the resonant frequency of the narrow strip). To keep the electrical wall boundary at the symmetrical plane, a narrow strip with width of 0.3 mm is applied in the prototype fabrication. Indeed, the numerical simulations show the influence of the metal strip width on the differential mode transmission can be neglected as long as the metal strip is not too wide.

To validate the aforementioned idea, we have conducted numerical simulation and experimental measurements on the differential spoof SPPs T-lines with narrow strip resonators. In simulations, open boundary condition is used to eliminate the reflection from environment, and the energy losses from dielectric and metal are considered. The simulated distributions of the electric fields (*E*_y_) at frequency of 3.5 GHz for common mode and differential mode are presented in Fig. 4(b) and (c) respectively. To manifest the effect of the narrow metal strip on electric field of the common mode, the simulated electric-field patterns of *E*_y_ for differential plasmonic arrays with and without the metal strip are shown in Fig. 4(b). It is visualized clearly that the electric field of the common mode is distorted by the metal strip. On the contrary, the electric field patterns of differential mode remain almost the same for plasmonic structures with and without metal strip, as shown in Fig. 4(c).

**Figure 5:**
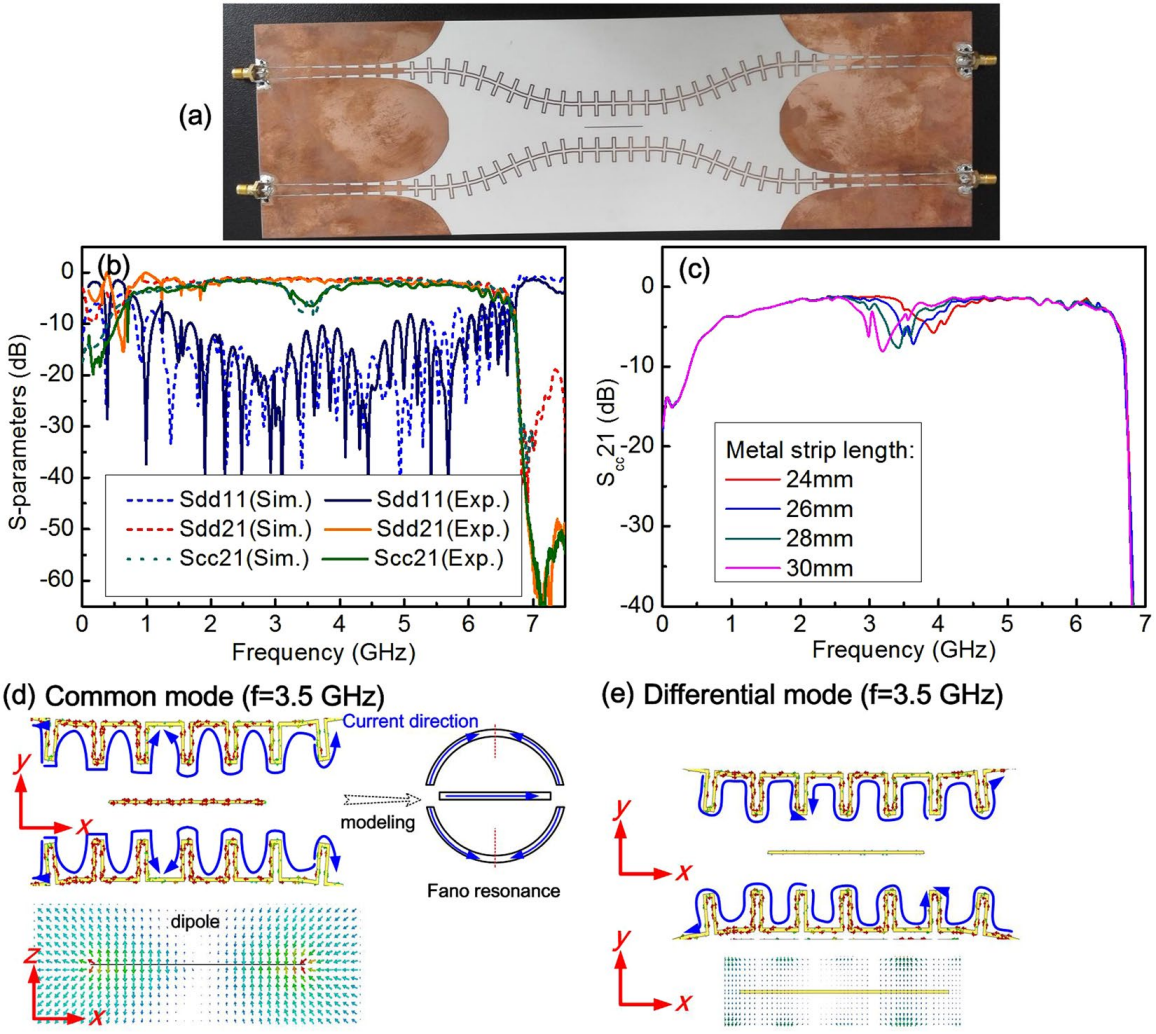
(**a**) The photo of the prototype sample. **(b)** Comparisons of simulated S-parameters with experimental results for differential mode and common mode. **(c)** Shift of the common mode rejection frequency with metal-strip length. **(d)** The current flowing on the strip and SPPs T-lines, and the Fano resonance like model for common mode suppression. **(e)** The current flowing on the strip and SPPs T-lines and electric field distribution around the strip in differential operation, which shows no resonance occurring in the central metal strip.

The photo of the differential SPPs T-lines is shown in Fig. 5(a) and the measured S-parameters are shown in Fig. 5(b) along with the simulated ones for comparison. It is observed the measured *S*_dd_11 is less than −10 dB in frequency range of 1.7~4.5 GHz, and *S*_dd_21 is about −1.1 dB ± 0.1 in frequency range of 2.2 GHz to 3.8 GHz. Here, *S*_dd_11 and *S*_dd_21 are the reflection coefficient and transmission coefficient of differential mode respectively. It is also noted that the transmission coefficient *S*_cc_21 and reflection coefficient *S*_cc_11 of common mode are almost the same as those of the differential mode in the frequency range of interest except for the notch of the common mode. The experimental *S*-parameters are agreement with simulations for the sample has 28 mm length of the loaded strip. Accordingly, the common mode notch occurs at 3.5 GHz and the rejection level is about 6.5 dB as can be observed in the curve of the common mode transmission coefficient *S*_cc_21. Furthermore, the common mode rejection frequency can be tuned by the strip length, while differential mode transmission remains unchanged, as shown in Fig. 5(b). A Fano resonance^24, 25^ like characteristic is observed in the *S*_cc_21 curve. An explanation is given as follows. For common mode, the in-phase *x* components of the TM polarized surface wave induces surface current flowing in *x* direction on the central strip. As a result, the strip acts as a dipole or multipole and resonates with the incident EM field when the strip length is approaching to the half wavelength of the SPPs, i.e. ${L}_{{\rm{strip}}}=N\cdot {\lambda }_{{\rm{spp}}}/2$, where ${L}_{{\rm{strip}}}$ is the strip length, ${\lambda }_{{\rm{spp}}}$ is the wavelength of the SPPs, and *N* is an integer. The length dependence resonance of the central metal strip determines the rejection frequency of common mode. On the other hand, the surface current cannot be invoked on the central strip by the anti-phase *x* components of EM field with the identical amplitude in differential mode operation (Fig. 5(d)). Consequently, the differential mode transmission is not affected by the metal strip.

**Figure 6:**
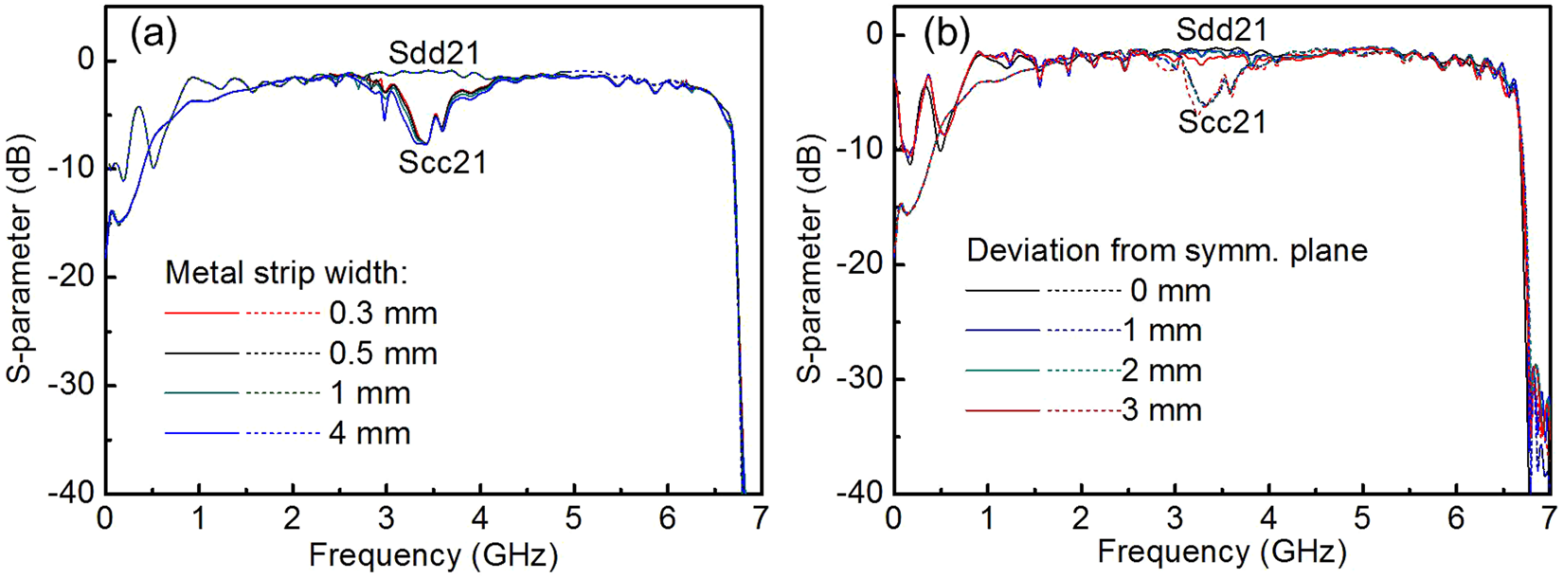
The influence of fabrication mismatch on the S-parameters caused by **(a)** the metal strip width and **(b)** the location deviation.

Finally, numerical simulations are carried out to evaluate the influence of the fabrication mismatches on the EM transmission in the differential SPPs T-lines. The effect of the metal strip width on the S-parameters is shown in Fig. 6(a). It is observed the influence of the metal strip width on the EM transmission can be neglected if the metal strip is symmetrical about the central symmetrical plane and not too wide. Since the strip width being far less than the half of wavelength (${\lambda }_{spp}/2$), the incident electric field cannot stimulate the surface current on the metal strip in width direction. As aforementioned, the orientation of the induced dipole is in the lengthwise direction and the resonance is mainly decided by its length as aforementioned. Therefore, a little variation in the metal strip width should have no obvious effect on EM wave transmission. The influence of metal strip deviating from the symmetric plane on EM transmission is shown in Fig. 6(b). While the metal strip shifting from the geometry center with a distance from 0 mm to 1 mm, the S-parameters almost keep unchanged. But as the metal strip deviates further from the symmetrical plane, the requirement for symmetricity by virtual ground boundary is not met and thus leads to scattering on the differential mode transmission. Considering the general machining accuracy in PCB is up to 0.1 mm nowadays, a little fabrication deviation in the strip width and location can be acceptable.

## Discussion

In this work, a new spoof SPPs transmission line has been constructed by arranging unit cells with open-cross shape in periodical array. The simulated results show the proposed open-cross array can provide enlarged propagation constant and hence enhanced confinement at metal/dielectric interface as compared to the conventional designs. Furthermore, a differential transmission line pair has been built with such two close plasmonic arrays. The common-mode rejection at specific frequency is achieved by placing a narrow metal strip at the symmetrical plane of the two SPPs waveguides. The notch frequency for common mode can be adjusted by tuning the metal strip length of the resonator while differential mode propagation remains unaffected. Both simulated and experimental results have been given, showing good agreement. The proposed high-performance spoof SPPs would be attractive in the applications of microwave/millimeter-wave circuits and devices with either single-ended or differential topology.
